# Administration of Santonin

**Published:** 1890-06-21

**Authors:** 


### THERAPEUTICS.

### ADMINISTRATION OF SANTONIN.

It is well known that unpleasant effects, even convulsions, have followed the administration of santonin in the case of adults as well of children, and since the anthelmintic action desired is a local one,—*i.e.*, on the worms, not on the patient—Dr. Lewis, of Berlin, recommends that it should be given in a form as little likely to be absorbed as is possible. He suggests olive or castor-oil as a suitable medium, with one of the ethereal oils, themselves actively injurious to the lower organisms, as a useful adjuvant.

